# Hypoglossal Nerve Stimulator Lead Extrusion: Successful Management and Reimplantation

**DOI:** 10.1002/oto2.70169

**Published:** 2025-09-18

**Authors:** Iman Adibi, Arman Saeedi, Alyssa N. Calder, Ryan Nord

**Affiliations:** ^1^ Department of Otolaryngology–Head and Neck Surgery Virginia Commonwealth University School of Medicine Richmond Virginia USA

**Keywords:** device extrusion, hypoglossal nerve stimulation, reimplantation, TYRX antibiotic pouch

Hypoglossal nerve stimulation (HNS) is an Food and Drug Administration (FDA)‐approved treatment for patients with moderate to severe obstructive sleep apnea (OSA) who are intolerant to continuous positive airway pressure (CPAP). The system includes a respiratory sensing lead, an implantable pulse generator (IPG), and a stimulation lead that together promote respiratory‐synchronized tongue protrusion during sleep. While the safety and efficacy of HNS are well‐documented, device complications are relatively rare but may require removal or revision surgery.^1^

## Case Report

A 74‐year‐old male with a body mass index of 26.0, hypertension, chronic obstructive pulmonary disease, insomnia, and moderate OSA (apnea‐hypopnea index [AHI] 15.4) presented to the otolaryngology clinic after 2 years of CPAP intolerance. His sleep regimen included temazepam, and his social history revealed smoking one pack per day and four daily alcoholic drinks. His drug‐induced sleep endoscopy revealed complete anteroposterior collapse of the velopharynx, partial lateral collapse of the oropharynx, partial tongue base collapse, and no collapse of the epiglottis, and thus he was deemed an excellent candidate for HNS.

He underwent successful HNS implantation utilizing the two‐incision technique. The patient's postoperative course was uneventful with successful device activation 1 month later. One‐month post‐activation, he reported cessation of snoring and improved restfulness. Three months post‐device activation, the patient presented after noticing wires protruding through his chest skin for the past week. He denied erythema, warmth, pain, drainage, or fever. Physical examination confirmed wire extrusion along the medial aspect of the chest incision without purulence. After a detailed discussion, the patient agreed to surgical exploration with wound revision and possible explant.

**Figure 1:**
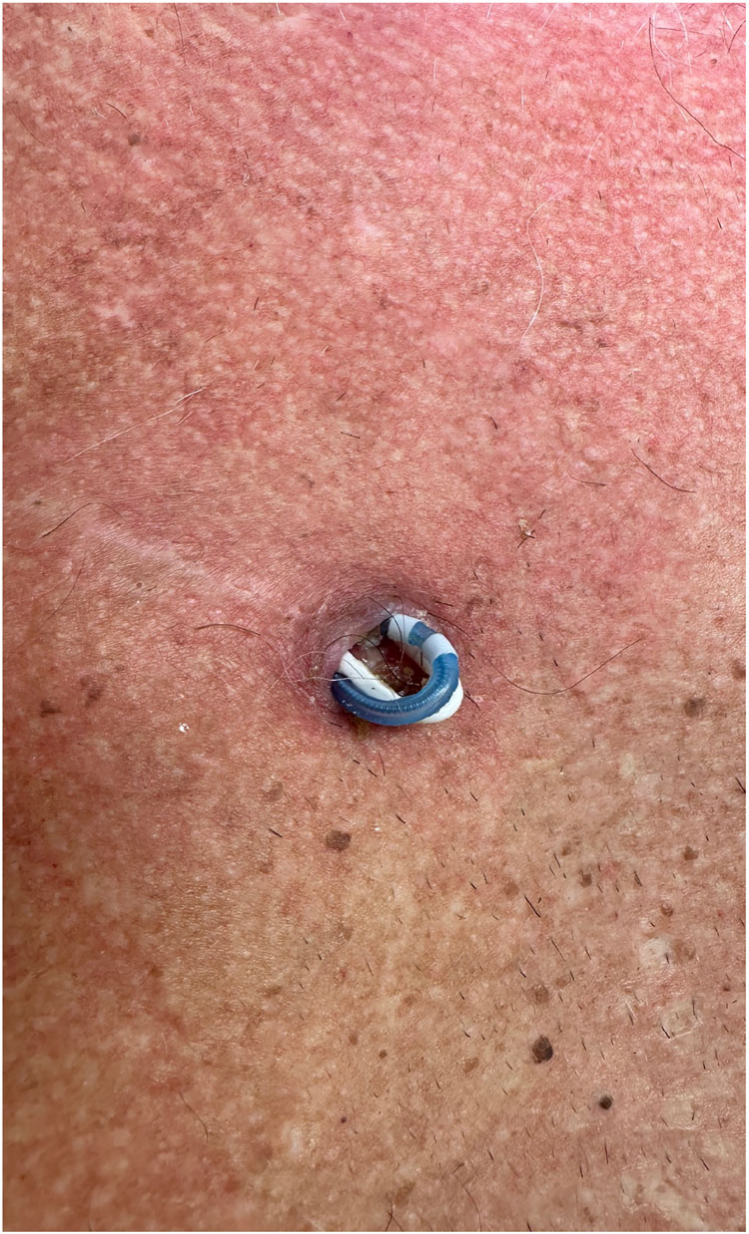
Extruded sensing and stimulation leads from the medial aspect of the chest incision.

**Figure 2:**
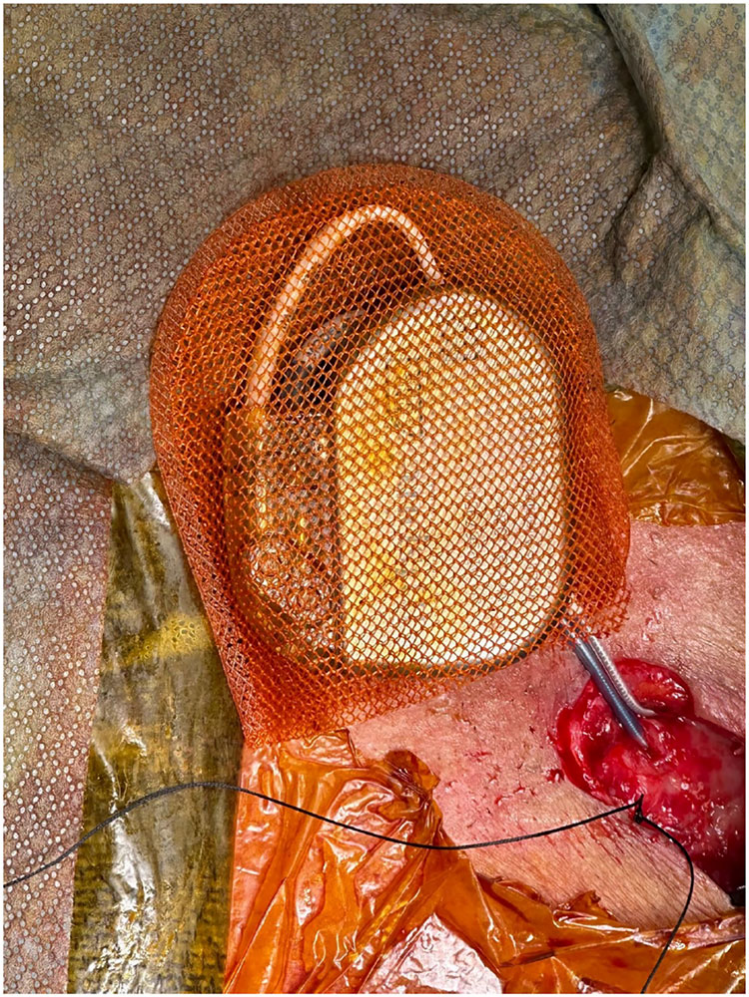
Implantable pulse generator placed in the TYRX antibacterial pouch for device reimplantation.

Intraoperative findings revealed extrusion of the sensing and stimulation leads, with approximately 2 cm of wire visible at the medial edge of the prior chest incision (Figure 1). First, pulsed saline lavage using an Interpulse (Stryker) Device was performed directly over the extruded hardware. Next, the chest incision was opened, and as more hardware was exposed, it was irrigated similarly. The IPG capsule was opened and found to be clean without infection. The suprapectoralis capsule was opened, the IPG was removed, and the wound pocket was again thoroughly irrigated without evidence of infection. The IPG and previously extruded proximal stimulation leads were placed in a medium TYRX (Medtronic) absorbable, antibacterial pouch and reimplanted in the suprapectoralis pocket (Figure 2). It was secured to the pectoralis fascia with 2‐0 silk sutures. The dehisced incision edges were excised to freshen the wound and closed in a multilayered fashion.

Recovery was uneventful. At 11‐ and 31‐day follow‐up, the patient denied fever, chills, or tenderness at the surgical site. The incision remained intact and appropriately healed at 77 days. He remained HNS compliant and denied snoring. This case report was deemed exempt by the Virginia Commonwealth University Institutional Review Board.

## Discussion

To our knowledge, this is the first reported case of HNS device extrusion from the chest incision with successful reimplantation. HNS device extrusion is rare, with an incidence rate of 0.21% and accounts for 9.2% of HNS‐related adverse events according to the FDA Manufacturer and User Facility Device Experience (MAUDE) database.^1^ Previous reports have documented HNS device extrusion through the pharynx and neck incision.^2^, ^3^ Unlike those cases, this patient presented with extrusion through the chest incision.

Reimplantation was successful without signs of infection, paralleling the neck extrusion case, where the device was also salvaged.^3^ Had there been signs of infection on exploration, the device would have been removed. Key steps likely contributed to preventing infection: extensive pulsed lavage, betadine prep, and TYRX antibacterial pouch use. The TYRX pouch, which releases minocycline and rifampin into surrounding tissue for a minimum of 7 days, has not been reported for HNS use but has been shown to significantly reduce the risk of cardiovascular implantable device infections and, therefore, can be considered in select HNS cases requiring salvage.^4^

The etiology of extrusion in this case remains unclear. The patient's regular cigarette and alcohol use, known risk factors of impaired wound healing, may have contributed.^5^ This case underscores the importance of patient education regarding modifiable risk factors postimplantation.

## Conclusion

This case represents the first report of HNS device extrusion from the chest incision and demonstrates that in the absence of infection, timely surgical management with the use of a TYRX pouch can result in successful reimplantation.

## Author Contributions

**Iman Adibi**, writing–original draft preparation; **Arman Saeedi**, writing–reviewing and editing; **Alyssa N. Calder**, writing–reviewing and editing; **Ryan Nord**, writing–reviewing and editing, supervision.

## Disclosures

### Competing interests

Ryan Nord: Paid consultant for Inspire Medical Systems, the manufacturer of the device studied in this research. Paid consultant for Medtronic. All other authors have no conflicts of interest to disclose.

### Funding source

None.
